# Magnesia as a Surgical Dressing

**Published:** 1874-01

**Authors:** 


MAGNESIA AS A SURGICAL DRESSING.—The interest which attaches to the use of dry earth as a surgical dressing in connection with the recent paper by Addinell Hewson and Conner, on this subject, has induced Dr. Ohlmeyer of Weissenburg to record his experiences in the use of the carbonate of magnesia in somewhat similar cases. He has found it of value—

1. In atonic ulcers. 2. In cases where the epidermis was eroded and the subjacent tissues were the seat of pain, and were prone to subsequent suppuration. 3. In relieving the pain, of inflamed wounds. 4. In cases where it was desirous to stimulate the affected surface, prevent the access of air, and limit the formation of pus. He was led, in the first instanee, to try this remedy, from its well-known action in those states of the stomach where there is an excessive formation of acids. These latter uniting with the base, magnesia, are neutralized, and carbonic acid evolved. Accordingly he believes that in exposed surfaces where the process of healing is prevented by fermentative action, this dressing is indicated. The use of it was attended with satisfactory results. The magnesia unites with the acids which form on the surface; it excludes the oxygen, forms an artificial covering, irritates the granulations, and forms a barrier against external and harmful agents.

In preparing the application he selects a fluid that will not readily oxidize. Oil answers this indication, and the kind he employs is the oil of sweet almonds. Adding to this the carbonate, he makes a tolerably fluid paste of salve. This is then spread upon linen and laid over the wound. It is held in place in the ordinary way.

Dr. Ohlmeyer also adds that he has used the carbonate successfully in facial erysipelas, when it was important to protect other patients from infection. In this latter case he used water as a substitute for oil.

